# A survey of community members' perceptions of medical errors in Oman

**DOI:** 10.1186/1472-6939-9-13

**Published:** 2008-07-29

**Authors:** Ahmed S Al-Mandhari, Mohammed A Al-Shafaee, Mohammed H Al-Azri, Ibrahim S Al-Zakwani, Mushtaq Khan, Ahmed M Al-Waily, Syed Rizvi

**Affiliations:** 1 Department of Family Medicine and Public Health, College of Medicine and Health Sciences, Sultan Qaboos University, P. O. Box 35, PC 123, Oman; 2 Department of Pharmacy, Sultan Qaboos University Hospital, P.O. Box 38, PC 123, Oman

## Abstract

**Background:**

Errors have been the concern of providers and consumers of health care services. However, consumers' perception of medical errors in developing countries is rarely explored. The aim of this study is to assess community members' perceptions about medical errors and to analyse the factors affecting this perception in one Middle East country, Oman.

**Methods:**

Face to face interviews were conducted with heads of 212 households in two villages in North Al-Batinah region of Oman selected because of close proximity to the Sultan Qaboos University (SQU), Muscat, Oman. Participants' perceived knowledge about medical errors was assessed. Responses were coded and categorised. Analyses were performed using Pearson's χ^2^, Fisher's exact tests, and multivariate logistic regression model wherever appropriate.

**Results:**

Seventy-eight percent (n = 165) of participants believed they knew what was meant by medical errors. Of these, 34% and 26.5% related medical errors to wrong medications or diagnoses, respectively. Understanding of medical errors was correlated inversely with age and positively with family income. Multivariate logistic regression revealed that a one-year increase in age was associated with a 4% reduction in perceived knowledge of medical errors (CI: 1% to 7%; p = 0.045). The study found that 49% of those who believed they knew the meaning of medical errors had experienced such errors. The most common consequence of the errors was severe pain (45%). Of the 165 informed participants, 49% felt that an uncaring health care professional was the main cause of medical errors. Younger participants were able to list more possible causes of medical errors than were older subjects (Incident Rate Ratio of 0.98; p < 0.001).

**Conclusion:**

The majority of participants believed they knew the meaning of medical errors. Younger participants were more likely to be aware of such errors and could list one or more causes.

## Background

In spite of the high reported rates of medical errors in various health care systems [1-5], most studies of medical errors focus on either analysing incidents reported or assessing health care professionals' views [6-9]. Furthermore, given the importance of using health care consumers' opinions and attitudes [10-12], few studies have assessed attitudes of health care users with regard to medical errors [13-17]. Some of these studies have found out that consumers have increased expectations as well as an awareness of their rights and responsibilities [18,19]. The importance of assessing consumers' views is demonstrated by the significant positive associations between satisfaction and improved compliance and continuity of care which ultimately leads to better outcomes, reduced rates of disease complications and the side effects of medications [20,21].

Knowledge about medical errors by health care consumers should help to strengthen health care provision and improve clinical practice [22]. Such knowledge could re-enforce the level of trust in health care systems in general and of professionals in particular. This is especially important given the publicity the media allocate to medical errors [23] as well as how the media play in modifying patients' health seeking behaviour [24,25].

Furthermore, having data from health care recipients facilitates proper community education programs about medical errors that enable patients differentiate between side effects, normal course of a disease and adverse events resulting from medical errors. This is particularly applicable to elderly and illiterate patients who, for example, may not appreciate the difference between a medical error and the side effect of a medication. In addition, these programs may help providers educate patients about the role the individual and the system in the development of an error, thus reducing blames on doctors as a main source of errors. In addition, such programs would also help improve reporting of medical errors by consumers [26]. Ultimately, consumers can have an active role in the quality of their own health care delivery, as partners rather than as passive users.

Oman is a developing country located on the south-eastern tip of the Arabian Peninsula with a population of 2.24 million based on the 1993 census [27]. The Gross Domestic Product per capita income (GDP) was estimated to be 11,466 U.S Dollars in the year 2005 [28]. The health services in Oman are funded by the government and provided free for all Omanis and non-Omanis working in the government sector. The standards of health services are equivalent to the industrialized nations. In the year 2005, the crude death was 2.53 per 1000 population, the infant mortality rate was 10.28, the under-five mortality rate was 11.05 per 1000 live-birth and the life expectancy at birth was 74.28 years [27]. However, despite such improvements many Omanis travel to other countries seeking health care. This might reflect a trend that deserves an exploration of its causes such as lack of trust on safety of care delivered.

Despite the benefits of exploring health care consumers' attitudes to medical errors, not much is known from developing health care systems. The objectives of this study were to assess health care consumers' perceptions of medical errors and to further examine factors affecting such perceptions. This study will be a starting point for further research in the field of patient's safety in Oman.

## Methods

The study was conducted in the North Al-Batinah region of Oman, from 15–26 January 2005. Two villages were selected because of close proximity to the College of Medicine and Health Sciences, Sultan Qaboos University (SQU), Muscat, Oman. All houses (250) in these two villages were included in the study. However, only 212 interviews took place (85% of the total) because some were unoccupied (families had moved away).

Data were collected using face-to-face interviews with the paternal head of the family. When the father was not at home, the eldest member (either male or female) over 18 years of age was interviewed. Interviews were carried out by third and fourth year medical students as part of their Village Health Care course in the College of Medicine and Health Sciences. These students had been trained in a 3-day course on community surveys and face-to-face interviews. To assure data quality, all student interviews were supervised by Family and Community Medicine doctors.

The questionnaire was developed after literature review, discussion with colleagues and pilot testing (by the medical students in their own village communities). The questionnaire was composed of three sections. Section one assessed demographic and other data (including age, sex, education, marital status, family income, usual source of health care, frequency of health care facility usage, history of chronic illnesses, and of any regular doctor appointments). Section two assessed participant's perceived knowledge about medical error definition ("Do you know what is meant by medical error?"). To follow up the participant's understanding, those who answered "Yes" were asked for at least one definition. Answers were then reviewed and coded into five categories: the prescription of wrong medications, the wrong diagnosis, a doctor's technical incompetence, technical incompetence of other staff and other examples such as staff attitude. These answers were then compared to our study definition. Subsequently, selection of answers falling under that definition was made. Section three had questions on related issues such as experience of medical errors and of its consequences. Answers to questions in section three vary between "Yes/No" format to selection of answers from a list of options. The study protocol was approved by the Medical Research and Ethics Committee of the College of Medicine and Health Sciences, SQU.

### Statistical Analysis

For categorical variables, frequencies and percentages were recorded. Differences between groups were compared using Pearson's χ^2 ^or Fisher's exact tests (for cells that have less than 5 responses). For continuous variables, means and standard deviations (± SD) were calculated. Differences between groups were analysed using Student's t-tests. The distribution of medical errors follows a Poisson distribution or one of its variants. One of the rarely met assumptions of a Poisson model is that the mean must equal the variance. When the *conditional *variance is greater than the mean, an over-dispersed model may occur producing incorrect variance estimates that are biased downwards. When this occurs, a negative binomial model, which does not constrain the *conditional *variance to equal the mean, is preferred over a Poisson Model [29,30]. Since there was significant over-dispersion, as denoted by the likelihood ratio test (p < 0.001), the association between the perceived knowledge on medical errors definition and age was analysed using the negative binomial model.

The associations between knowledge and various predictors were analysed using univariate and multivariate logistic regressions. The dependent outcome variable was the perceived knowledge of the meaning of medical error. Covariates included age, gender, educational level, marital status and family income.

The multivariate logistic model was examined extensively to evaluate overall model fit and any assumptions. The overall fit was assessed using the Hosmer & Lemeshow goodness-of-fit statistic [31] and the area under the Receiver Operating Curve (ROC) [32]. The Hosmer & Lemeshow statistic analyses the actual versus the predicted responses; theoretically, the observed and expected counts should be close. Based on the χ^2 ^distribution, a Hosmer & Lemeshow statistic with a *p*-value greater than 0.05 is considered a good fit. The ROC curve is a graph of the sensitivity against one minus specificity as the threshold cut-off is varied, and also calculates the area under the curve. The ROC curve provides a measure of the model's discriminatory power. A model with perfect prediction has an ROC of 1.0; an area of 0.5 provides no better discrimination than chance. Models with area under the ROC curve of greater than 0.7 are preferred. A *priori *two-tailed level of significance was set at the 0.05 level. Statistical analyses were performed using STATA version 10.0 software.

## Results

About half of the participants were male (53%; n = 112). The overall mean age was 34 ± 13 years with an age range from 15 to 94 years, literacy was 83% (n = 177) and 70% (n = 148) were married. Ninety three per cent (n = 197) stated that they had visited health care facilities (primary or secondary care) over 5 times a year, which included visits for curative/preventive services (e.g. vaccination). The average number of visits per person per year was 10.2 compared to the Ministry of Health (MoH) figures (an average of 4.4 per person per year in 2005) [27]. The discrepancy between rates is because the current study counted accompanying someone as a visit, compared to MoH statistics which count only visits for individual health care services. Forty six percent (n = 97) reported a history of chronic illness such as diabetes mellitus or recurrent low back pain. In 2005 non-communicable diseases represented 54.5% and 39.8% of outpatient and inpatient morbidity respectively [27]. Fifty eight percent (n = 124) stated that they saw their regular doctor on most visits.

Questioned about understanding of "what is meant by medical error", 78% (n-165) responded "Yes". Of these, 34% to referred to wrong medication, 26.5% to wrong diagnosis, 13% to wrong operations and 4% to wrong injections. Interestingly, around 23% of the definitions given were referring to causes of medical errors rather than exact definition. These were related to professionals and patients such as lack of doctor's experience and not following doctor's advice (Table 1).

**Table 1:** Participants' perceived definitions of medical errors

**Serial No.**	**Definition categories**	**Definitions given by participants**	**Number (%)**
1	Wrong prescription/dispensing of medication	Wrong medication	93 (34)
2	Wrong diagnosis	Wrong diagnosis	73 (26.5)
3	Doctors' technical in-competence	Wrong surgery	36 (13)
		Technical incompetence*	10 (3.6)
		Lack of doctor's experience*	7 (3)
4	Other staff technical in-competence (nurses and pharmacist)	Giving wrong injection	11 (4)
		Pharmacist incompetence*	4 (1)
5	Others	Errors by doctors	5 (2)
		Forgotten surgical items	2 (0.7)
		Wrong vaccination	2 (0.7)
		IV canula left in site for 25 days	1 (0.4)
		Error in first aid	1 (0.4)
		Wrong BP reading	1 (0.4)
		Wrong procedure	1 (0.4)
		Doctor ignorance*	14 (5)
		Poor staff attitude*	4 (1)
		Not updating patients*	2 (0.7)
		Faulty equipment*	1 (0.4)
		Doctors overload*	1 (0.4)
		Slowness in giving care*	1 (0.4)
		Nurses ignorance*	1 (0.4)
		Wrong information by the patient+	1 (0.4)
		Not following doctors advise+	1 (0.4)
		Intake of un-prescribed medicine+	1 (0.4)
		Intake of herbal medicine+	1 (0.4)

**Total**			**275**

Percents are out of total number (275). Please notice that some participants gave more than one definition.

* Causes of medical errors

+ Patient-related factor

Associations between perceived knowledge of medical error and various predictors were evaluated using both univariate and multivariate logistic regression models. The overall multivariate logistic regression model was statistically significant (LR χ^2^(7) = 35.61; p < 0.001) and it accounted for 15.9% of the variance in perceived knowledge of medical errors definition (Pseudo R^2 ^= 15.88). The model fits reasonably well. The Pearson's χ^2 ^goodness-of-fit statistic, using 10 near equal-size groups as suggested by Hosmer & Lemeshow, was 1.66 and the *p*-value was 0.990. The ROC curve was 0.76. The model correctly classified 81% of the cases.

Age was negatively correlated with perceived medical error knowledge. This was significant in both univariate and multivariate regression models (Table 2). The older participants were less likely to be knowledgeable about medical errors. Specifically, after controlling for other variables, each year increase in age was associated with a 4% reduction in participant's perceived knowledge of medical error definition (CI: 1% to 7%; p = 0.045). There was a trend in both the univariate (OR 0.33; CI: 0.14 to 0.79; *p *= 0.012) and multivariate (OR 0.45; CI: 0.15 to 1.31; *p *= 0.144]) regression models for married participants to be less knowledgeable than their unmarried counterparts; this did not attain statistical significance in the multivariate logistic model (Table 2). There was a positive relationship between family income and perceived knowledge of medical error definition; the higher the family income, the more knowledge on its definition was seen. This trend was seen in both the univariate and multivariate regression models (Table 2).

**Table 2:** Univariate and multivariate logistic regression models (N = 212).

**Independent Variable**	**N**	**Univariate**		**Multivariate**	
			
		**Odds Ratio [95% CI]**	**p-value**	**Odds Ratio [95% CI]**	**p-value**
Age	212	0.94 [0.91–0.96]	<0.001	0.96 [0.93–0.99]	0.045
Male gender	112	0.63 [0.32–1.22]	0.169	0.82 [0.36–1.85]	0.629
Educational level					
Illiterate	35	Ref			
Preparatory	147	4.20 [1.92–9.19]	<0.001	1.70 [0.61–4.77]	0.314
Secondary & above	30	8.50 [2.17–33.3]	0.002	1.58 [0.28–8.88]	0.603
Married	148	0.33 [0.14–0.79]	0.012	0.45 [0.15–1.31]	0.144
Family income					
<200	66	Ref			
200–500	102	2.19 [1.08–4.43]	0.029	1.99 [0.87–4.55]	0.104
>500	44	5.35 [1.70–16.8]	0.004	4.73 [1.37–16.4]	0.014

N = Number of participants in each category; CI = Confidence Interval; the variables were entered into the multivariate logistic regression model simultaneously; p-values were generated using both univariate and multivariate logistic regression models

Using exploratory univariate statistics, other variables such as source of healthcare, frequency of healthcare use, history of chronic illness, and seeing one doctor regularly were found to have no significant effect on awareness of medical errors definition. These variables were excluded from the final logistic models (Table 3). Of those who believed they knew what was meant by medical error (n = 165), 49% (n = 80) had had experience of medical error in the preceding year, either by themselves or a family member. The outcomes included severe pain (44%; n = 36), substantial loss of time at work/school or other activities (19%; n = 15), disability (15%; n = 12) and death (9%; n = 7). The nature of the experience was diagnostic errors for 32 participants (40%); 21 (26%) and 22 (28%) of the participants stated that errors were due to surgical and medication errors, respectively. Ninety-five participants (58%) of those who believed they knew what a medical error was, felt that medical errors occurred often in the community, compared to 7 participants (4%) who felt that errors never happened.

**Table 3:** Socio-demographic and educational variables of the study participants stratified by perceived knowledge of medication error definition (N = 212).

**Characteristic**	**Knowledge of Medication Errors**		
	
	**No **(n = 47)	**Yes **(n = 165)	**p-value**
Age, mean ± SD, in years	43 ± 17	31 ± 11	<0.001
Age category, n (%)			
15–24 years	5 (11%)	47 (28%)	<0.001
25–34 years	10 (21%)	67 (41%)	
35–44 years	11 (23%)	27 (16%)	
>44 years	21 (45%)	24 (15%)	
Gender, n (%)			
Female	18 (38%)	82 (50%)	0.167
Male	29 (62%)	83 (50%)	
Educational Level, n (%)			
Illiterate	17 (36%)	18 (11%)	<0.001
Reads & writes/preparatory	27 (57%)	120 (72%)	
Secondary and above	3 (6%)	27 (16%)	
Marital Status, married, n (%)	40 (85%)	108 (65%)	0.010
Family Income, n (%), in OR			
<200	23 (49%)	43 (26%)	0.004
200 – 500	20 (43%	82 (50%)	
>500	4 (9%)	40 (24%)	
Usual Source of Healthcare, n (%)			
Local Health Center	33 (70%)	108 (65%)	0.272
Local Hospital	6 (13%)	20 (12%)	
Private Hospital	7 (15%)	37 (22%)	
Others (e.g. Traditional Healer)	1 (2%)	0 (0%)	
Frequency of Healthcare Use, n (%)			
1–5	2 (4.3%)	13 (7.9%)	0.787
6–10	21 (45%)	72 (44%)	
>10	24 (51%)	80 (48%)	
History of Chronic Disease, n (%)	22 (47%)	75 (45%)	0.869
Seeing a Doctor Regularly, n (%)	26 (55%)	98 (59%)	0.617

SD = Standard deviation; Percents are column percents; OR = Omani Rials; Differences between groups were analyzed using *Student's *t-test, *Pearson's *χ^2 ^test, and *Fisher's Exact *test whenever appropriate.

With regard to the causes of medical error for those who believed they knew its meaning (n = 165), 48.5% of the participants (n = 80) felt that uncaring health care professionals was the main cause (Table 4). Lack of training of health care professionals was identified as the next most frequent cause (46%; n = 76). Forty two percent of the participants (n = 70) appreciated that work overload was one of the causes of medical errors. A further 39% (n = 64) of the participants considered lack of time spent with the patient as the cause. When asked to list other causes of medical errors, none of the participants listed patients' factors although they listed some factors when asked to define medical error. It was also found that younger participants were more able to list one or more possible causes of medical errors compared to older participants. Specifically, each one year increase in age was associated with a 2% reduction in the likelihood of listing one or more possible cause of medical error (Incident Rate Ratio of 0.98: CI 0.97 to 0.99; p < 0.001).

**Table 4:** Participant responses to a list of causes of medical errors

**Cause**	**Number (%)**	
	
	**Yes**	**No**
Uncaring health care professional	80 (48.5)	85 (51.5)
Lack of training	76 (46)	89 (54)
Work overload	70 (42)	95 (58)
Lack of time spend with the patient	64 (39)	101 (61)
Shortage of doctors	57 (34.5)	108 (65.5)
Poor handwriting	37 (22)	128 (78)
Poor supervision	33 (20)	132 (80)
Complexity of medical care	26 (16)	139 (84)
Shortage of paramedical	24 (14.5)	141 (85.5)
Shortage of nurses	21 (13)	144 (87)
Lack of computerized medical record	11 (7)	154 (93)

## Discussion

To our knowledge this is the first study in Oman to assess health care consumers' perceptions about medical errors. This study shows that the majority of participants believed they knew what is meant by the term 'medical error'. In Oman, the issue of medical errors is currently publicly discussed through newspapers, television and radio programs, actively encouraged by the Shura Council (State Consultative Council) and the Ministry of Health. This could explain increased community members awareness about medical errors. This finding is in line with what was found by Gallagher et al, that all participants were aware of the topic of errors in medicine [33]. However, Blendon et al found that 68% of the public were unaware of the meaning of medical errors [22]. The majority of participants being young and literate in the present study could explain our findings. Interestingly, other authors distinguished medical errors resulting from failures of a planned action (i.e. errors of execution) to those due to the wrong management plan (i.e. errors of planning) [34]. Participant statements not falling under the above definition were not considered as definitions of errors (Table 1). For example, statements such as given wrong medication, making wrong diagnosis or performing wrong surgery were considered as definitions of errors, whereas statements such as lack of doctor's experience and technical incompetence were considered as causes of errors.

However, in the present study it was found that 23% of the definitions refer to causes of medical errors rather than the exact definition. Such a finding is similar to what was found by Van Vorst and colleagues in their study which showed that at least 41% of the 180 reported mistakes received were not judged to be medical mistakes when coded with a taxonomy designed to specifically describe medical errors [17].

This finding reflects the need to communicate with the community about the definition of medical error and its causes. Furthermore, it reflects the role health care professionals, mainly physicians, play in educating patients about investigations done and their results, diagnosis, medication/s they are taking and their side-effects. This may help community members to differentiate between an error and a side-effect of medication or a complication resulting from the normal course of a disease. Ultimately, this would improve patients' reporting of such outcomes, thus enable health care providers take needful actions.

It is of interest to note that multivariate logistic regression showed an association between perceived knowledge of the meaning of medical error and age. Such associations could have two explanations. On the one hand, younger patients might be more likely to ask health care professionals for an explanation of events compared with their older counterparts. In addition, younger patients might have more knowledge on issues such as health care safety, thus empowering them to raise questions about their own care. However, our results could be linked to the findings of patient satisfaction studies which show that elderly patients are more satisfied with their health care provision than younger or more educated patients, regardless of the quality of care provided [35].

Forty-nine percent of the participants in our study stated that they had an experience with a medical error, either themselves or with one of their family members. This rate is similar to that reported by Blendon et al, in which 42% of the participants or their family members experienced a medical error [22]. In contrast, a community survey about medical errors carried out by the European Commission showed that 23% of the patients or their family member had encountered a medical error [36]. Another study found that 22% of the patients stated that they or family members had experienced medical errors of some kind [37]. Furthermore, a large percentage of participants in the current study felt that errors were common. These rates reflects concerns among health care consumers that deserves consideration by health care systems such as the need to explore these experiences more and link the results with those of clinical audits [37,38]. This will help providers identify strengths and weaknesses of their health care systems and plan for improving patient safety. Vigilance in these areas will ultimately help health care systems gain the trust of the communities they serve.

Participants listed work overload (for health care professionals) and lack of time physicians spend with their patients as very important causes of medical errors. This finding is similar to that of a study which showed that physicians' stress, fatigue, overwork and inadequate time with their patients to be at the top of the causes of medical errors [22]. These factors affect the doctor-patient and doctor-health professionals communication, and the educational role doctors ought to play. This reflects the need to look at these factors and reduce the communication gap among health care professionals and patients as it has been shown that gap in communication was a common cause of medical errors [39].

Despite our participants' perceived knowledge of the meaning of medical error and estimates of the prevalence in the community, none of them listed patient factors as causes. This may reflect the lacking of medical knowledge from patients' side to describe medical errors, particularly related to its causes [17]. Furthermore, health care consumers may not be aware of their own role in health care delivery or they may have assumed that only the health care system was being studied. This ultimately affects satisfaction with the quality of care delivered, because blame is directed to health care providers and institutions, thus affecting trust. These observations further re-enforce the passive role patients assume in their own health care, forgetting that patients can be experts in their own care and can thus play a major role in reducing medical errors such as adverse drug reactions [40,41]. This might then indirectly affect the health care system; patients might not follow physicians' recommendations, ultimately leading to a vicious cycle in which all drug side-effects or all disease complications may be assumed to be medical errors. Such findings reflect an essential need to educate the community members about the role individual patients and the system play in the development of medical errors. This would help in reducing the pressure on health care professionals either from the public or the media when it comes to medical errors.

Lack of patient education about these as well as other causes of medical errors could be due to the defensive nature of many health care systems when medical errors are discussed [42,43]. However, this can be set against the high preferences of patients towards disclosure of errors and the provision of more information about the underlying disease shown by some studies [33,44]. Although one study showed that many people interviewed thought that patients were often at least partially responsible for errors in their health care, the public were less likely (than physicians) to attribute errors to patient factors [22]. Participants in the current study were not explicitly asked about their role in medical errors and were left to comment in answers to open questions.

This study has limitations. The first is that there was no independent verification that someone in the family suffered a medical error. However, health care consumers are now more oriented towards modern medicine with regard to their rights and responsibilities. Secondly, reliability and validity tests were not performed on the questionnaire, however, one could argue that the questionnaire was in fact not a proper survey tool, and hence would not require these tests to be conducted. Thirdly, the convenience sampling of the population in the two villages located close to the College of Medicine and Health Sciences, Sultan Qaboos University (SQU) could have affected the generalizability of our sample cohort. A larger study comprising the different areas of Oman is warranted to corroborate these findings.

## Conclusion

This study shows a majority of respondents believe they know what is meant by the term 'medical error'. Younger people are more likely to believe this than older people. Therefore, given the high rate of chronic illness and increased use of health care facilities by elderly people, more health education programmes should be directed to the older community members. These programmes should aim to increase awareness about the possible medical errors that might occur in health care delivery. Ultimately, this will help to differentiate between unfortunate drug side-effects and medical errors. Furthermore, a large percentage of the definitions given were referring to the causes of medical errors rather than exact definition. In addition, no participant raised patient factors as contributory causes to medical errors. There needs to be further education to increase patients' awareness about the meaning of a medical error and its causes and of patients' own active roles in the health care delivery. Finally, community surveys about medical errors should be supported by clinical audits in order to show the exact prevalence of medical errors in the system.

## Competing interests

The authors declare that they have no competing interests.

## Authors' contributions

ASA–M, MAA–S, MHA–A and MK participated in the design of the study, data collection and in drafting the manuscript. ISA–Z, AMA–W and SR performed the statistical analysis, and interpretation of data. ASA–M, MHA–A and ISA–Z revised the manuscript. All authors have read and approved the final manuscript.

## Pre-publication history

The pre-publication history for this paper can be accessed here:


